# Transcatheter Arterial Embolization for Alleviating Chronic Musculoskeletal Pain and Improving Physical Function: A Narrative Review

**DOI:** 10.3390/diagnostics13010134

**Published:** 2022-12-30

**Authors:** Bow Wang, Keng-Wei Liang, Chia-Hui Chen, Chien-Kuo Wang

**Affiliations:** 1 Department of Medical Imaging, National Cheng Kung University Hospital, College of Medicine, National Cheng Kung University, Tainan 704, Taiwan; 2 Department of Medical Imaging, Chung Shan Medical University Hospital, Taichung 402, Taiwan; 3 School of Medicine, Chung Shan Medical University, Taichung 402, Taiwan; 4 Institute of Medicine, Chung Shan Medical University, Taichung 402, Taiwan; 5 Department of Medical Imaging and Radiological Sciences, I-Shou University, Kaohsiung City 824, Taiwan

**Keywords:** transarterial embolization, genicular artery embolization, angiogenesis, musculoskeletal disorder, chronic pain, osteoarthritis, tendinopathy, inflammation

## Abstract

Chronic musculoskeletal pain imposes immense suffering and diminishes the quality of life for millions of patients worldwide; the pain persists despite the use of standard conservative treatments. Increases in our understanding of the pathophysiological mechanisms underlying musculoskeletal disorders indicate the involvement of inappropriate angiogenesis. Accordingly, the resulting neovessels are the target of emerging treatments for chronic musculoskeletal pain, including transarterial embolization. The use of this noninvasive procedure to treat pain refractory to standard therapy in a variety of musculoskeletal conditions is the focus of numerous recent investigations. Here, we describe the pathophysiological indications for the use of transarterial embolization and summarize the findings of studies investigating its use in a variety of histopathological conditions and anatomical sites.

## 1. Introduction

Chronic pain is a common and challenging problem faced by clinicians and patients [1]. Chronic musculoskeletal pain arises from disorders involving bones, muscles, ligaments, tendons, and joints, and is experienced by an estimated 20–33% of people worldwide [2]. A wide variety of musculoskeletal disorders can lead to chronic musculoskeletal pain, most commonly causing low back pain, neck pain, as well as rheumatory- and osteoarthritis (OA)-associated joint pain. The primary presenting symptom in patients with chronic musculoskeletal disorders is intense, localized pain, which is often accompanied by body aches, malaise, and stiffness [2]. Chronic musculoskeletal pain can lead to reduced activity, sleep disturbances, fatigue, mood alterations, and severe disability [3]. Inadequately managed musculoskeletal pain has harmful impact on quality of life, causing a significant socioeconomic burden [2].

Typically, chronic musculoskeletal pain was first treated with conservative managements, such as exercises specific to the pain source, weight loss if needed, education, and the use of oral or topical non-steroidal anti-inflammatory drugs [4]. In cases recalcitrant to conservative measures, further pharmacological and surgical treatment options may be available depending on the particular etiology. The number of suitable pharmacological options is increasing. For example, anabolic growth factors, cathepsin K inhibitors, and Wnt inhibitors are able to arrest structural progression; nerve growth factor inhibitors can reduce OA pain [4]. After exhausting all currently available treatment options, some patients continue to experience chronic musculoskeletal pain; therefore, the development of new treatment strategies for pain control in these patients is of utmost urgency [1].

Transarterial embolization (TAE) is an established minimal invasive procedure used to occlude target blood vessels by infusing an embolic agent for a wide range of conditions. It is most commonly used to control bleeding, or to decrease the size of hypervascular tumors through selective “embolization” of small arteries [5,6,7,8,9]. Notably, TAE techniques have been applied to treat a variety of painful musculoskeletal conditions due to abnormally chronic inflamed or hypervascular tissues stimulating angiogenesis [10,11,12,13]. The abnormal angiogenetic neo-vessels, accompanied by pain-related nerve fibers ingrowth were identified; after blocking the target angiogenesis by TAE, the pathologic tissue including the nerve fibers was then involuted. Furthermore, three recent systematic reviews also concluded that TAE is a safe therapeutic option without major complications for patients with chronic join pain [12,13,14].

Okuno et al. were the first group to describe their successful treatment experience of TAE on tendinopathies and enthesopathies, published in 2013 [15]. Since then, plenty of studies have reported on the role of TAE as a potential pain management on various chronic inflammatory musculoskeletal conditions. Herein, we describe the pathophysiological indications for the use of TAE and summarize the findings of studies investigating its use in various histopathological conditions and anatomical sites.

## 2. Mechanisms Underlying Chronic Musculoskeletal Pain Associated with OA

Chronic OA-associated musculoskeletal pain results from pathophysiological processes involving angiogenesis, neurogenesis, and inflammation. Angiogenesis, the growth of new blood vessels, occurs in both the bone and synovium in osteoarthritic joints [16]. The degree of osteochondral vascularity in OA correlates with the severity of cartilage changes and disease progression [17], indicating the critical involvement of angiogenesis in OA joint pathology. This new blood supply to normally avascular tissues in the affected joint facilitates the invasion of inflammatory cells [18], which in turn promotes cartilage destruction and chronic local inflammation [12]. Of inflammatory cells, activated macrophages release a variety of proangiogenic factors (vascular endothelial growth factor and β-nerve growth factor), thereby stimulating synovial angiogenesis and nerve growth [16]. Thus, aberrant angiogenesis in OA boosts inflammation, disrupts the osteochondral junction, and promotes abnormal sensory nerve growth in vulnerable joint structures [19]. It was suggested that blood vessels and nerves abnormally outgrow from subchondral bone into articular cartilage that is normally avascular and aneural [20], is responsible for the pathogenesis of joint pain [19]. Therefore, angiogenesis at the osteochondral junction is an attractive target for therapeutic intervention [16].

One such intervention involves blocking the blood flow in osteochondral neovessels via TAE. This promising treatment option for symptomatic OA has demonstrated to be safe and efficacious for use in a variety of joints, particularly, in the knee. It was suggested in 2015 that genicular artery embolization (GAE), a minimally invasive intra-arterial intervention, was able to effectively alleviate refractory chronic pain and restore knee function in patients with knee OA [21]. Numerous subsequent studies of GAE for knee OA have provided mounting evidence in support of this finding. Interestingly, further studies suggest that TAE is effective for treating OA pain in joints other than the knee and musculoskeletal pain associated with other inflammatory conditions. The following sections review the use of TAE in a range of joints and conditions.

## 3. Osteoarthritis

### 3.1. Knee Osteoarthritis

Although initially designed to treat knee hemarthrosis, GAE has been recently and widely adapted to treat symptomatic knee OA [22]. Chronic knee pain is a major symptom of knee OA. While arthroplasty is needed to alleviate pain and other symptoms in late-stage knee OA, GAE has proven successful for the non-operative management of early-stage knee OA symptoms [23]. GAE involves selective embolization of geniculate branches, depending on the site of knee pain [21]. Briefly, ipsilateral antegrade femoral artery access was created using 3–5 French sheath. In addition, a 3–5 French angiographic catheter, with or without a 1.7-, 2.0-, or 2.4 French microcatheter, was used to identify the target genicular vessel(s) with abnormal staining or blush-type enhancement on the arterial phase, where was the location of reported pain. Embolic agent diluted with iodinated contrast was administered in small increments until blood flow was stagnated. Embolic agents used for GAE include imipenem/cilastatin, Embozene, resorbable microspheres, and polyvinyl alcohol. However, a recent meta-analysis of GAE for knee OA showed no difference in efficacy between embolic agents [24]. GAE efficacy is commonly evaluated using the Visual Analog Scale (VAS), a pain rating scale, and the Western Ontario and McMaster Universities Osteoarthritis Index (WOMAC), an assessment of pain, stiffness, and physical functioning in ordinary daily activities.

The consensus of multiple studies of GAE for knee OA is that GAE safely and effectively reduces knee OA pain in patients with mild to moderate OA recalcitrant to conservative treatment [25,26,27,28,29]. No major complications were reported; the most common minor complication was transient erythema in the region of embolization, which would be resolved within 1–3 weeks [14]. Several studies investigating the effects of GAE on physical functioning, as assessed by the WOMAC score, report significant early and long-term improvements at 6 months [27] and 1 year [28,29] after embolization.

The severity of knee OA as rated using the Kellgren–Lawrence (KL) scale clearly affects GAE outcomes. Studies comparing GAE outcomes between patients with mild to moderate knee OA (KL grade 1–3) and those with severe knee OA (KL grade 4) show a strong correlation between KL grade and pain reduction after GAE [25,30]. While patients with mild to moderate knee OA experienced significant and long-lasting pain reduction after GAE, patients with severe knee OA had a brief 1-month decrease in pain severity followed by a gradual increase back to the original severity score within 3 months after GAE [25]. Another study found that in addition to high KL grade, large bone marrow lesions (BMLs) and severe meniscal injuries revealed by magnetic resonance imaging (MRI) indicated poor responses to GAE [30].

Taken together, these findings indicate that GAE is efficacious for treating knee OA in patients with mild to moderate but not severe knee OA. MRI has been recommended for determining the suitability of GAE in individual cases, as it provides information regarding the status of soft tissues within the knee that KL grading does not [30].

### 3.2. Trapeziometacarpal Osteoarthritis

Trapeziometacarpal osteoarthritis (TM-OA), also recognized as carpometacarpal osteoarthritis, is osteoarthritis at the base of the thumb; the prevalence of TM-OA is approximately 15% in adults aged 30 and over [31]. TM-OA can be extremely debilitating and frustrating because it constrains thumb opposition [32]. As with OA in other joints, inflammation and angiogenesis are implicated in disease progression. To address whether TAE is a suitable treatment option for TM-OA, a cohort of 31 patients with TM-OA resistant to conservative management was infused with imipenem/cilastatin into the radial artery [33]. The technical success rate was 100%, and assessments of pain and disability showed significant improvements over baseline through 24 months after treatment, with no major adverse events [33].

### 3.3. Finger Osteoarthritis

Finger OA is often occurred in the distal interphalangeal joint, which is highly prevalent among women over the age of 50 [34]. Conservative treatments can provide only temporary pain relief [35]. In a single case study, a woman with recalcitrant OA in 3 fingers of her right hand was treated with radial artery injection of imipenem/cilastatin [36]. Her VAS score decreased immediately after arterial embolization and kept decreasing over the course of 1 month to zero in 6 months. Her swelling and stiffness partially improved. The authors concluded that infusion of embolic agents directly into the radial artery has durable efficacy for treating distal interphalangeal OA of the hand [36].

### 3.4. Facet Joints and Sacroiliac Joints Osteoarthritis

Low back pain is the most frequently observed musculoskeletal problem worldwide [37]. Although surgical treatment is appropriate for low back pain of neuroanatomical origin, an estimated 85–90% of patients have non-specific low back without identified pathoanatomical cause [37]. Treatment of these patients therefore focuses on reducing pain and its consequences [37]. Unfortunately, the effective therapies for non-specific lower back pain are still under investigation [38]. The lumbar facet joint and sacroiliac joint are the most frequently observed sources of non-specific low back pain [39], and the involvement of low-grade inflammation [40] prompted an investigation of TAE for pain relief. In a cohort of 14 patients with recalcitrant chronic low back pain, clinical success of TAE was found in 79% and 57% of patients at 3 and 24 months after TAE, respectively [39]. Pain and disability assessment scores were significantly improved at 1, 3, and 24 months after TAE, with no major adverse events [39].

### 3.5. Shoulder Osteoarthritis

Reports of the use of TAE for treating OA in the shoulder are rare in the literature. In a study of TAE for pain relief, one of the three patients in the cohort had shoulder osteoarthritis [41]. This patient experienced significant pain relief within the first day after intervention, but long-term follow-up data are not reported.

### 3.6. *Osteoarthritis-Related synovitis*

**Figure 1:**
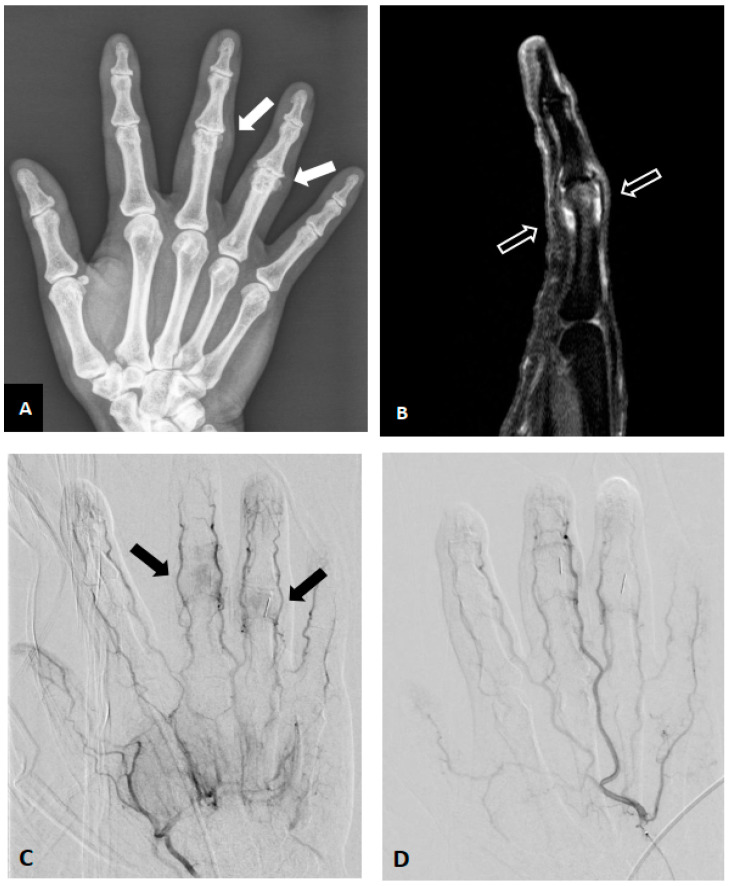
Representative pre- and postprocedural images of the right hand 3rd and 4th proximal interphalangeal (PIP) joint osteoarthritis in a 50-year-old female with chronic pain and swelling. The results of all rheumatoid profile blood tests were within the normal ranges. (**A**) Right hand plain film revealed joint space narrowing with spurs formation (white arrows). (**B**) Preprocedural sagittal MRI (fat-saturated T1-weighted post-contrast) of the 3rd finger revealed synovium thickening and enhancement at approximately the 3rd PIP joint (blank arrows). (**C**) Preprocedural angiography of the right ulnar artery showed angiogenesis (black arrows) overright 3rd and 4th PIP joints. (**D**) Postembolization angiography via ulnar artery indicated elimination of angiogenesis after embolization. The patient’s joint pain and swelling both were improved after treatment.

Synovitis is common in OA joints [42], which can be also improved by endovascular embolization techniques. Synovitis was found in 74% of patients with various grades of knee OA, and was strongly correlated with the radiographic severity of knee OA [43]. Assessment of structural OA features using whole-organ MRI at 2 years after GAE showed significant improvement in synovitis compared to baseline, without osteonecrosis or aggressive progression of degenerative changes, in patients with mild to moderate refractory knee OA [26]. On the other hand, two cohort studies found that approximately 94% of patients with hand OA had synovitis in at least one finger joint [44,45]. As indicated in Figure 1, the inflamed (swollen) synovium of the proximal interphalangeal joint was ameliorated after radial artery injection of imipenem/cilastatin in a woman with recalcitrant OA in three fingers of her right hand [36].

### 3.7. Osteoarthritis-Related Bone Marrow Lesion

Advanced knee OA is characterized by cartilage destruction and pathophysiological changes in the underlying subchondral bone. One of these changes is subchondral bone marrow lesion (BML), also recognized as bone marrow edema. BMLs arise from mechanical overloading of subchondral bone that contains fatigue fractures, or an increased bone turnover due to OA [46]. Detectable on MRI, the presence of BMLs in knee OA is associated with weight-bearing pain [47]. Additionally, BML size correlates with the degree of cartilage destruction and associated pain [48]. Thus, BMLs in knee OA have become the target for treatment strategies to alleviate knee pain and possibly enhance the understanding of the pathogenesis of knee OA [46].

**Figure 2:**
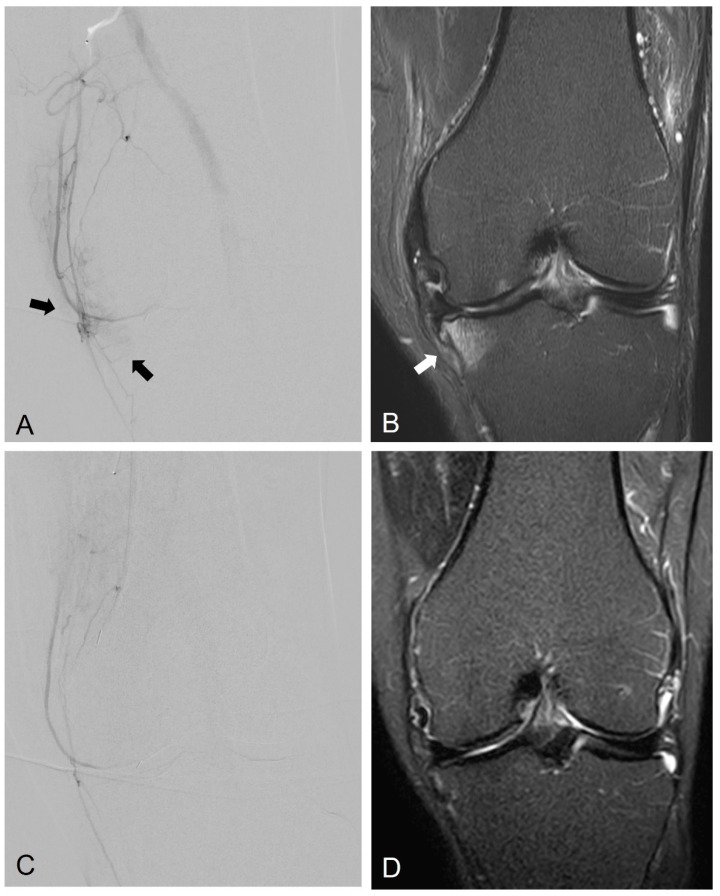
Representative pre- and postprocedural images of the right knee in a 69-year-old male with KL grade 2 osteoarthritis with associated pain. (**A**) Preprocedural angiography of the right descending genicular artery showed angiogenesis over medial aspect of the knee joint (black arrows). (**B**) Preprocedural coronal MRI (fat-saturated T2-weighted TSE) revealed subchondral bone marrow lesion over right tibia plateau (white arrow). (**C**) Postembolization angiography indicated elimination of angiogenesis after GAE. (**D**) Follow-up MRI acquired 3 months after GAE revealed the reduction of subchondral BML. The patient’s knee pain and function were improved after GAE.

Although GAE has been widely used to treat symptomatic knee OA, studies on the effect of GAE on bone BMLs are rare [30,49]. A recent unpublished retrospective study reports that GAE is efficacious for treating knee OA with or without BML, and that GAE significantly decreased the size of BMLs in OA patients without osteonecrosis [50]. The representative images indicating the reduction of subchondral bone BML after GAE are shown in Figure 2.

Several lines of evidence also suggest the importance of BMLs in OA diagnosis and treatment [51] and in OA pathogenesis [52]. Pharmacological treatments specifically targeting BMLs not only decreased BML size but also improved knee pain and function [53,54], suggesting the potential of such BML-specific therapeutic approach in the treatment of knee OA [54]. As the presence and size of BMLs correlate with the disease severity of knee OA [55], the use of MRI to identify and characterize BMLs is important for thorough disease assessment to determine appropriate treatment for OA [50]. Although the clinical significance of BMLs in knee OA was demonstrated [56], the application of BML-specific treatment for treating OA in other joints remains to be investigated.

## 4. Non-Osteoarthritic Synovitis

### 4.1. Adhesive Capsulitis (Synovium)

Adhesive capsulitis, also called as frozen shoulder, is featured by a gradual onset of shoulder pain and constrained shoulder motion [57]. The pathophysiological mechanisms underlying adhesive capsulitis are not fully understood yet, but the involvement of chronic inflammation, fibroblastic proliferation, angiogenesis, synovitis, as well as fibrotic contracture of the rotator interval was suggested [57].

Evidence that newly-grown blood vessels and nerves are possible sources of pain has prompted investigations of TAE for treating adhesive capsulitis resistant to conservative treatment [58]. A rat model of frozen shoulder model demonstrated that neovascularization was developing after injury, and TAE reduced the growth of blood vessels and inflammatory changes in the injured area and restored physical function [59].

**Figure 3:**
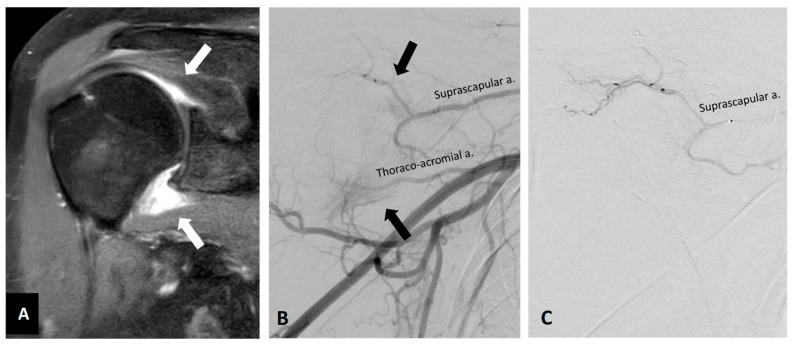
Representative pre- and postprocedural images of the right shoulder adhesive capsulitis in a 56-year-old female with chronic pain and limited range of motion. (**A**) Preprocedural coronal MRI (fat-saturated T1-weighted post-contrast) revealed synovium and capsule enhancement (white arrows). (**B**) Preprocedural angiography of the right axillary artery showed angiogenesis (black arrows) over the corresponding enhanced area of MRI. (**C**) Postembolization angiography via thoracoacromial artery and other arteries indicated the elimination of angiogenesis after TAE. The patient’s pain and range of motion were improved after TAE.

An ongoing clinical trial including 40 patients with adhesive capsulitis reports promising midterm results, with a significant decrease in pain and a substantial improvement in mobility during flexion and abduction observed at 6 months after embolization [57]. Similar results are reported in a study of 24 patients undergoing TAE for adhesive capsulitis [60] in which assessments at 1, 3, and 6 months after TAE showed that VAS pain scores significantly decreased and American Shoulder and Elbow Surgeons (ASES) scores, an assessment of pain and functional limitation in the shoulder, improved significantly. An earlier study in a small cohort of 7 patients with adhesive capsulitis confirmed the presence of abnormal neovessels at the rotator interval in all 7 patients, and also reported a fast decline in nighttime pain scores 1 week after TAE and further improvements at 1 and 6 months [58]. Similarly, another ongoing trial observed hypervascularity in all 20 patients with adhesive capsulitis and reports significant improvements in VAS and ASES scores at 1, 3, and 6 months after TAE [61]. All of these studies demonstrated the efficacy of TAE for the treatment of adhesive capsulitis refractory to conservative treatment. The representative images of the right shoulder adhesive capsulitis before and after TAE are shown in Figure 3. The enhancement of rotator interval and axillary joint capsule on contrast-enhanced MRI indicates synovitis and capsulitis, which are essential features for diagnosis of adhesive capsulitis [62,63].

### 4.2. Secondary Adhesive Capsulitis (Post-Operation)

Secondary adhesive capsulitis typically presents after an injury or surgery. Although primary adhesive capsulitis typically involves loss of motion in all directions, secondary adhesive capsulitis often involves a more defined loss of motion that affects some movements but not others. As with primary adhesive capsulitis, the pathogenesis of this condition likely involves angiogenesis, suggesting that TAE may effectively treat patients with recalcitrant pain. Indeed, abnormal vessels were observed in 20 of 25 patients with secondary adhesive capsulitis, and TAE treatment resulted in significant pain reduction and mobility improvement at 6 months [64].

### 4.3. Hip Synovitis

Only one study has investigated the use of TAE for treating hip synovitis [65]. In this case study of one patient with a 10-year history of recalcitrant hip pain due to hip synovitis, the branches of the ascending branch of the lateral femoral circumflex artery were embolized using microbeads [65]. Four months after the procedure, the pain was relieved and walking ability was improved. The authors emphasize technical challenges in identifying and accessing the target vessels and caution against the use of microbeads in the hip due to the risk of necrosis [65].

## 5. Tendon and Enthesopathy

### 5.1. Shoulder Tendinopathy

Tendinopathy, a general term for disorders involving tendons, includes two distinct conditions: tendinitis and tendinosis [66]. While tendinitis is an acute inflammatory condition often caused by small tears in the tendon, tendinosis is a chronic non-inflammatory degeneration of tendon collagen due to chronic overuse [66]. The involvement of angiogenesis and accompanying nerve growth in tendinosis suggests the potential use of nerve- or vessel-targeted treatments [67].

Data regarding tendinosis treatment using TAE are scant, and the cohorts in the few studies investigating such treatment are heterogeneous with respect to diagnosis [68,69] and treatment site [15]. A cohort study evaluated the efficacy of TAE in 13 patients with shoulder or elbow tendinopathy, including calcific tendinitis, rotator-cuff tendinopathy, and lateral epicondylitis, reports a significant decrease in VAS pain score at 1 week and 4 months after TAE [68]. However, conclusions regarding TAE efficacy for shoulder tendinopathy cannot be determined because the data were analyzed for the total cohort, regardless of etiology [68]. Another study of TAE for shoulder pain included 15 patients with adhesive capsulitis and/or tendinobursitis [69], while the results show a clinical success rate of only 20%, etiology was not considered in the data analysis [69]. Further studies in homogeneous cohorts are needed to determine the efficacy of TAE for treating specific shoulder tendinopathies.

### 5.2. Lateral Epicondylitis

**Figure 4:**
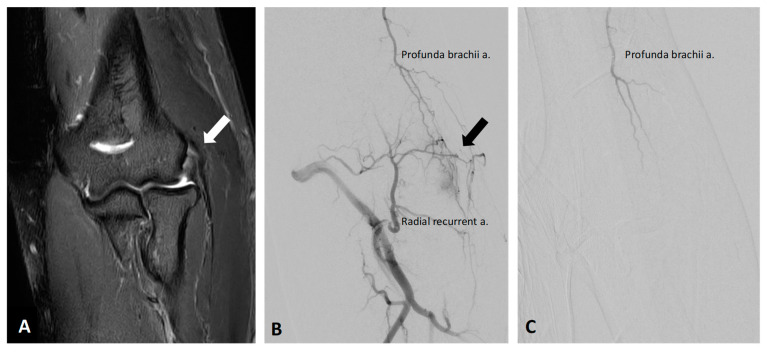
Representative pre- and postprocedural images of the right elbow lateral epicondylitis in a 58-year-old male. (**A**) Preprocedural coronal MRI (fat-saturated T2-weighted image) revealed abnormal thickening with elevated signal intensity within the common extensor origin from the lateral epicondyle (white arrows). (**B**) Preprocedural angiography of the right radial recurrent artery showed angiogenesis with reflux to profunda brachii artery (black arrows) over the corresponding enhanced region of MRI. (**C**) Postembolization angiography via profunda brachii artery and other arteries indicated the elimination of angiogenesis after TAE. The patient’s lateral elbow pain and function both were improved after TAE.

Lateral epicondylitis, well-known as tennis elbow, is a degenerative angiofibroblastic tendinosis that causes pain in the outer side of the forearm from the elbow to the wrist [70]. A pilot study in a cohort of 22 lateral epicondylitis patients with moderate to severe pain observed abnormal neovessels in all participants, and reported that TAE significantly improved the VAS score, the score of the Patient-rated Tennis Elbow Evaluation Questionnaire, and the pain-free grip strength over a 2-year follow-up [70]. Moreover, MIR obtained 2 years after TAE revealed amelioration of tendinosis and improvement of tear scores [70]. Several studies report similar observations, although with less robust results due to heterogeneity in smaller cohorts [15,68]. The representative images of the right elbow lateral epicondylitis before and after TAE are shown in Figure 4.

### 5.3. Medial Epicondylitis

Medial epicondylitis, also called as golfer’s elbow, causes pain in the inner side of the forearm from the elbow to the wrist [71]. As with lateral epicondylitis, this condition is a degenerative tendinosis involving aberrant growth of new blood vessels and nerves. As such, several studies have investigated the efficacy of TAE in treating chronic recalcitrant pain in medial epicondylitis patients, although the number of studies and cohort sizes are small. In a cohort of 14 patients with medial epicondylitis refractory to conservative treatment, clinical success was achieved in 85.7%; the mean VAS pain score and Quick-DASH score, a measure of physical function and symptoms, decreased significantly over time from 1 day to 6 months after TAE [71], and these improvements lasted for up to 12 months after TAE in 9 out of 14 patients [71]. Another study with just one medial epicondylitis patient in the cohort showed that TAPE resulted in significant pain relief within the first day after intervention, but long-term follow-up data were not reported [41].

### 5.4. Patellar Tendinitis

Patellar tendinitis, commonly called as jumper’s knee, is an injury to the tendon connecting the patella to the tibia [72]. Repetitive jumping produces huge amount of energy in the extensor mechanism, which leads to tendinosis; and angiogenesis is likely involved in the pathogenesis of this degenerative disorder [72]. Embolization was demonstrated to effectively and safely reduce neoangiogenesis in a porcine model of patellar tendinitis, although the effect of embolization on pain was not investigated yet [73]. Although no clinical studies have addressed TAE treatment specifically for patellar tendinitis alone, two patients with patellar tendinitis have been included in studies of TAE in heterogeneous cohorts. In a study of TAE for pain relief, one of the three patients in the cohort had patellar tendinitis [41]. All three patients experienced significant pain relief within the first day after intervention, but long-term follow-up data are not reported. Another patient with patellar tendonitis was included in a heterogeneous cohort of seven patients; TAE significantly reduced VAS pain score in all seven patients [15].

### 5.5. Plantar Fasciitis

Plantar fasciitis, the most frequently observed cause of pain on the bottom of the heel, results from degenerative irritation of the plantar fascia and nearby perifascial structures [74]. Most patients can be managed non-surgically, whereas patient satisfaction is low and the recurrence of pain is frustrating [74]. Because few plantar fasciitis patients have been included in TAE studies, data regarding its efficacy in these patients is very limited. A case study of one plantar fasciitis patient reports the presence of abnormal vessels at the calcaneal attachment and severe recalcitrant pain before treatment [75]. Imipenem/cilastatin was administered directly into the posterior tibial artery twice at one month interval. The pain gradually decreased at 1 week after the first treatment, and the patient could engage in activities of daily living without difficulty at 3 months after the first treatment. The authors concluded that TAE of the posterior tibial artery is a feasible minimally invasive treatment option for plantar fasciitis [75]. Similar conclusion was reported in a study of TAE in a cohort that included one patient with plantar fasciitis [15].

### 5.6. Insertional Achilles Tendinopathy

Insertional Achilles tendinopathy is characterized by painful thickening of the Achilles tendon that is inserted on the posterior calcaneus [76]. In addition to progressive posterior heel pain, the typical symptoms include swelling, burning, and stiffness. Although most patients respond well to non-surgical management that reduces pressure and inflammation, surgical options may be still necessary in some cases. Preliminary evidence in a study of TAE in a heterogeneous cohort that included one patient with Achilles tendinopathy [15] shows that the procedure was technically successful in all patients, and TAE significantly reduced VAS score in all patients.

## 6. Others

### Trapezius Myalgia

Trapezius myalgia is characterized by acute or persistent neck and shoulder pain due to the presence of pain, stiffness, and tightness in the upper trapezius muscle [77]. Trapezius myalgia is most common among those who engage in repetitive manual work, often involving strained posture [77]. A study investigating TAE treatment in 42 patients with trapezius myalgia showed a significant decrease in pain and improvement in physical function during the 6 months following TAE, with a clinical success rate of 71.4% [78].

## 7. Conclusions

Taken together, the studies reviewed here constitute a body of evidence showing that TAE safely and effectively alleviates pain and improves physical function in patients with chronic recalcitrant musculoskeletal pain at a variety of anatomical sites. During TAE, if angiogenesis is not obvious, a small amount of embolic agents can still be administered, according to the patient’s pain site that was pre-procedurally marked on the skin using metallic marker, angiosome, and/or evoked pain [79]. Evidence also suggests the involvement of BMLs in the pathogenesis of knee OA, and further investigation of this line of evidence may broaden our understanding of the mechanisms underlying chronic musculoskeletal pain. The use of TAE to decrease the size of OA-associated BMLs has emerged as another potential treatment for chronic musculoskeletal pain in the knee.

## Data Availability

Not applicable.
